# The HELIX Project: Tracking the Exposome in Real Time

**DOI:** 10.1289/ehp.122-A169

**Published:** 2014-06-01

**Authors:** Carol Potera

**Affiliations:** Carol Potera, based in Montana, has written for *EHP* since 1996. She also writes for *Microbe*, *Genetic Engineering News*, and the *American Journal of Nursing*.

People are exposed to a multitude of environmental chemicals through air, water, food, and consumer products. A person’s total environmental exposure, acquired from conception to death, is called the “exposome.”^1^ The Human Early-Life Exposome (HELIX) project, a European collaboration, is an ambitious effort that will seek to characterize children’s exposomes as the children progress through early life.^2^ The six research stages of HELIX are described in this issue of *EHP*.^3^

HELIX, a project of 13 partner institutions, will measure environmental exposures of up to 32,000 mother–child pairs and their consequent impact on the growth, development, and health of the children. “Pregnancy and the early years of life are well recognized to be periods of high susceptibility to environmental damage with lifetime consequences. This makes early life an important starting point for development of the exposome,” says project coordinator Martine Vrijheid of the Centre for Research in Environmental Epidemiology in Barcelona, Spain.

**Figure:**
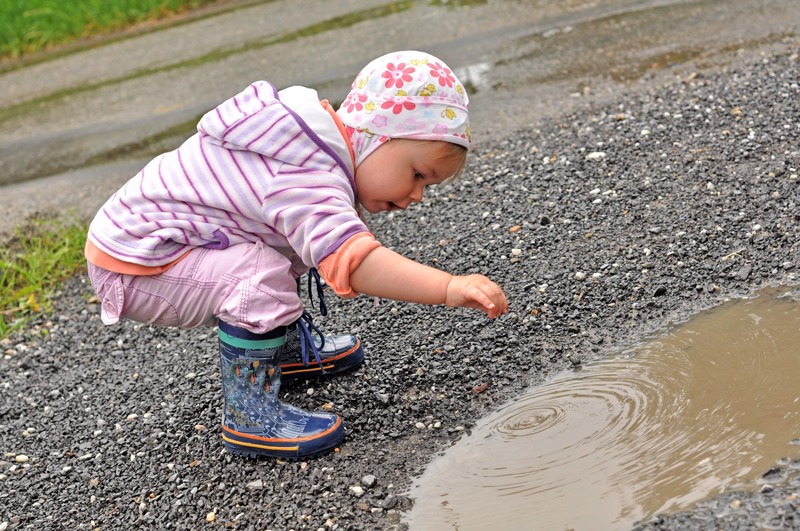
From conception onward the exposome becomes the record of every exposure a person has ever experienced. © Shutterstock/Marcel Jancovic

In 2005 cancer epidemiologist Christopher Wild coined the term *exposome* to describe the environmental counterpart of the genome. “At its most complete, the exposome encompasses life-course environmental exposures (including lifestyle factors), from the prenatal period onwards,” Wild wrote.^4^ He highlighted the need for complete assessments of environmental exposures in epidemiological studies of cancer. Today the exposome concept has been extended to other chronic diseases that carry large societal and economic costs.^1^

HELIX will use data from six ongoing, prospective European birth cohorts of mothers and children living in Spain, France, the United Kingdom, Norway, Greece, and Lithuania. Large amounts of health data already have been collected, which HELIX investigators will pool. They also plan to collect extensive biomarker data for a subset of 1,200 mother–child pairs.

External exposure measures for food, water, air pollution, pesticides, noise, and ultraviolet (UV) radiation will be integrated with molecular markers from metabolomic, proteomic, transcriptomic, and other “omic” studies. Then the investigators will estimate the burden of childhood disease from multiple environmental exposures. The HELIX project will run for 4.5 years.

The results are expected to help investigators identify unknown health hazards and benefits to better target future preventive measures and regulations. “Characterization of the exposome in early life can provide very effective tools for disease prevention, given that interventions at that time can reshape biological programming and shift the body’s developmental track to normal function,” says Vrijheid.

Among the innovative tools created specifically for HELIX is ExpoApp, a mobile application for tracking participants’ activity levels. ExpoApp uses GPS and a smartphone’s built-in accelerometer to track a person’s location and measure physical activity every 10 seconds. Participants will wear ExpoApp-enabled smartphones for a week, along with air pollution and UV monitors. The data will be used to calculate the amount of air inhaled and an individual’s exposure to air pollutants.

Because the exposome encompasses the progression of exposures over a lifetime, it’s an overwhelming research concept to understand and impractical to fully fund at one time, says Paul Lioy, a professor of environmental and occupational medicine at Robert Wood Johnson Medical School. HELIX “is a well-designed study and great first attempt to focus on one part of the exposome to demonstrate its proof-of-concept principles,” Lioy says.

The methods used to study the mothers and children participating in HELIX require many different tools that cut across multiple scientific disciplines. By publishing the complex study design of HELIX in *EHP*, Lioy says “other environmental health and exposure scientists may be inspired to develop parallel studies of smaller populations to validate other components of the exposome concept.”
